# Incidence and predictors of severe postpartum hemorrhage after cesarean delivery in South Central Ethiopia: a retrospective cohort study

**DOI:** 10.1038/s41598-023-30839-x

**Published:** 2023-03-03

**Authors:** Dereje Zewdu, Temesgen Tantu

**Affiliations:** 1 Department of Anesthesia, College of Medicine and Health Science, Wolkite University, Wolkite, Ethiopia; 2 Department of Obstetrics and Gynecology, College of Medicine and Health Science, Wolkite University, Wolkite, Ethiopia

**Keywords:** Health care, Medical research, Risk factors

## Abstract

Severe postpartum hemorrhage is an obstetric emergency that needs immediate intervention and is a leading cause of maternal death. Despite its significant health burden, little is known, about its magnitude and risk factors, especially after cesarean delivery in Ethiopia. This study aimed to evaluate the incidence and predictors of severe postpartum hemorrhage following cesarean section. This study was conducted on 728 women who underwent cesarean section. We retrospectively collected data from the medical records, including baseline characteristics, obstetrics, and perioperative data. Potential predictors were investigated using multivariate logistic regression analyses, adjusted odd ratios, and a 95% confidence interval to see associations. A p-value < 0.05 is considered statistically significant. The incidence of severe postpartum hemorrhage was 26 (3.6%). The independently associated factors were previous CS scar ≥ 2 (AOR 4.08: 95% CI 1.20–13.86), antepartum hemorrhage (AOR 2.89: 95% CI 1.01–8.16), severe preeclampsia (AOR 4.52: 95% CI 1.24–16.46), maternal age ≥ 35 years (AOR 2.77: 95% CI 1.02–7.52), general anesthesia (AOR 4.05: 95% CI 1.37–11.95) and classic incision (AOR 6.01: 95% CI 1.51–23.98). One in 25 women who gave birth during cesarean section experienced severe postpartum hemorrhage. Considering appropriate uterotonic agents and less invasive hemostatic interventions for high-risk mothers would help to decrease its overall rate and related morbidity.

## Introduction

Postpartum hemorrhage (PPH) is a leading cause of maternal mortality and morbidities, resulting in 27.1% of maternal deaths globally, varying from 13.4% in high-income countries to 34% in African countries^1,2^. The incidence of severe postpartum hemorrhage (PPH) is estimated to occur in 0.3–5% of all births: but markedly varies between geographic regions over the world, and its increasing rate is a growing public issue^3–13^. The presence of at least one of the following conditions—estimated blood loss > 1000–2500 mL, peripartum hemoglobin decline > 4 g/dL (compared to the baseline hemoglobin value measured before delivery), transfusion of at least four units of packed red blood cells, and hemostatic interventions—was considered to imply severe PPH.

While poorly managed PPH can rapidly progress to severe PPH and is strongly associated with an increased need for hysterectomy, unpredicted ICU admissions, and prolonged hospital stays, effectively managed PPH can prevent the occurrence of SPPH^14–16^. Besides, it may also represent a traumatic childbirth experience, resulting in long-term paternal and maternal psychological impact—postpartum depression and post-traumatic stress disorder^16–18^. Maternal death in sub-Saharan countries remains the highest in the world, and PPH is a crucial contributor to maternal mortality in these regions^19^. In Ethiopia, about 65% of maternal death occurred during the postpartum period^1,20^.

Certainly, the magnitude and predictors for PPH have been extensively investigated; however, little is known about the incidence of severe PPH and its predictors, particularly after cesarean delivery (CD) in Ethiopia and even in sub-Saharan countries^6,21^. Compared with normal birth, cesarean births, especially in labor cesarean section (CS) remain a common risk factor for severe PPH^4,7,22–24^. The need for peripartum blood transfusion in women undergoing CD is also higher^25^. However, the ability of the risk assessment methods to predict severe PPH in women who give birth particularly by CS is not well supported by the existing information.

Amongst studies specifically evaluating predictors for severe PPH after CD^24,26–28^, previous CS scar^26,27^, antepartum hemorrhage^24,27^, multiple pregnancies^23,28^, preeclampsia^3^, general anesthesia^23,24,28^, antepartum anemia^27,28^ and maternal age ≥ 35 years^3^ are reported as potential predictors; however, PPH may also occur in some women with no known risk factors^4^.

Given that, PPH remains a markedly underestimated global burden due to a lack of consensus concerning its definition and diagnosis^29^: uncertainties in the quantification of blood loss, varying definitions of severe PPH, and varying methodologies are responsible for the observed inconclusive finding between studies^30,31^. Further, clinical practices for preventing and managing PPH vary substantially depending on medical resources and the health system.

Despite the existing variation, this distinction is critical because the management of PPH after CS may differ from that of vaginal birth. After all, PPH is represented as a large volume of blood loss at CS as contrasted to vaginal birth, thus identifying risk factors for severe PPH after CD allows for early diagnosis and interventions that may prevent the development of further complications^32^.

Therefore, in this study, we aimed to determine the incidence of severe PPH after CD and identify its potential predictors at a specialized teaching hospital of Wolkite University, Southern Central Ethiopia. The findings of this study provide information for clinicians, researchers, and policymakers to maximize the quality of postpartum care and evidence-based intervention to decrease the incidence of severe PPH after cesarean section.

## Methods

### Study population and study design

This retrospective cohort study included all consecutive women who underwent cesarean delivery after 28 weeks of gestation in WKUSH between January 2019 and December 2022 (n = 728). This study was carried out under the Declaration of Helsinki Ethical Principles for Medical Research involving human subjects protocol^33^. Ethical approval was received from the Ethical Review Board of the institution with ethical clearance letter no. RCSUILC/55/22 and informed written consent were waived due to the retrospective nature of the study design.

### Sample size determination and sample technique

The sample size was not calculated; we included all eligible participants who underwent cesarean section during the study period using a consecutive sampling technique.

Data from 746 consecutive pregnant women, who underwent CS during the study period, were reviewed. Among them, while 728 (97.58%) women who met the eligibility criteria were included in the final analysis, 18 participants with incomplete medical records documentation were excluded.

### Data collection

Data were collected by two trained data collectors from the medical record, including prospectively collected baseline, obstetrics, and perioperative data of all mothers in the study hospital using a standardized checklist adapted from previous studies. A pre-test was performed on 5% of the sample size outside the study area.

Based on previous literature and clinical plausibility, we included a standardized set of information comprising pre-pregnancy information and clinical data on pregnancy-acquired and perioperative periods. The pre-pregnancy characteristics included maternal age, residency area, ANC visits, BMI, co-existing disease, and parity. Pregnancy-acquired characteristics included a previous history of abortion and abnormal uterine bleeding, current singleton or twin pregnancy, previous history of CD, prepartum anemia (< 11 g/dL), severe preeclampsia, eclampsia, HELLP syndrome, antepartum hemorrhage, induction/augmentation, and prolonged labor and perioperative data included types of CS, incision, types of anesthesia, birth weight, blood transfusion, and hemoglobin change. Hemoglobin difference was calculated by using hemoglobin values obtained during labor or within 24 h before CS and the postoperative period.

### Clinical practice

In our hospital, PPH was managed using pharmacological and surgical interventions, per the standard protocol. However, due to the inaccessibility of tranexamic acid, packed RBC, fresh frozen plasma, and/ or platelet concentrates in our setup; we mainly relied on whole blood, oxytocin, and misoprostol as a prophylaxis and treatment regimen for PPH. Blood transfusion was administered by anesthetists, resident obstetricians, midwives, and ward nurses.

The uterotonic agents used as prophylaxis for PPH included the administration of 10 IU of oxytocin intramuscularly, 0.2 mg of ergometrine, and 600 mcg of sublingual or rectal misoprostol, as part of the active management of the third stage of labor. Approaches to managing PPH, starting from oxytocin drip in saline solution, misoprostol, and blood transfusion, up to surgical interventions to arrest the PPH include uterine artery embolization, uterine compression sutures, exploratory laparotomy, subtotal, and total abdominal hysterectomy.

The diagnosis of PPH and severe PPH; is determined by the responsible obstetricians in charge depending upon an estimated blood loss, shock index, and level of hemoglobin drops. PPH diagnosis is by an estimated blood loss of more than 500–1000 ml, a hemoglobin decline of greater than 2 g/dl, or at least the need for two units of blood transfusions. Estimated blood loss of more than 1000–1500 ml, a hemoglobin reduction above 4 g/dl, or at least four units of blood transfusions are required to be considered severe PPH.

For the study purpose, we define severe PPH using hemoglobin difference between baseline and after CS, amount of whole blood transfused, and uterine hemostatic interventions, to minimize selection bias.

### Study outcome

The main outcome measure was severe PPH, which was defined as the presence of at least one of the following criteria: hemoglobin differences of ≥ 4 g/dl between baseline and post-delivery, transfusion of ≥ 4 units of whole blood cells due to significant hemodynamic deterioration, and if hemostatic interventions (compression suture, uterine or hypogastric artery ligation, hysterectomy) was performed within 24 h of CS delivery^7,27^.

### Operational definitions

Prepartum anemia is defined as a hemoglobin level of less than 11 g/dl using the last measurement on the day of cesarean section.

Baseline hemoglobin: the last measurement of hemoglobin level at least within 24 h before cesarean section.

Antepartum hemorrhage: any type of vaginal bleeding occurring from 28 weeks of gestation and before the birth of the baby.

Severe preeclampsia: defined as diastolic blood pressure ≥ 110 mm hg or the presence of severe sign, and/or proteinuria ≥ 5 g.

Preterm birth: birth occurring after 28 weeks and before 37 completed weeks of gestation.

### Statistical analysis

Data were checked, coded, and entered into SPSS (Statistical Package for the Social Sciences for Windows version 26.0, SPSS Inc., Chicago, Illinois, USA) for final analysis. Results were converted into categorical data and presented as frequency tables. The presence of an association between independent and dependent variables was checked using the Pearson chi-square test. All independent variables were analyzed using bivariate analysis, and the variables that had an association were entered into a multivariable logistic regression analysis, and a p‐value < 0.05 was considered to be a risk for severe PPH in this study. The results of associated variables were displayed as a frequency table, odds ratio (OR), and adjusted odds ratio (AOR) with a 95% confidence interval (CI).

### Ethics approval and consent to participate

Wolkite University's ethical review board offered ethical clearance with ethical clearance letter no.RCSUILC/55/22 and written consent was waived due to the retrospective nature of the study design.

## Results

### Baseline characteristics of participants

During the study period, of 746 women who underwent cesarean delivery, from ≥ 28 weeks of gestation, 728 (97.58%) were recruited for final analysis. Of these, the incidence of severe PPH was 26 (3.6%). While the residency area, co-existing diseases, BMI, and parity between those with and without severe PPH were comparable, the women with age ≥ 35 years, poor ANC follow-up (< 2 visits), and twin pregnancy were significantly high in the severe PPH group, [11.46% vs 2.37%; p-value < 0.001], [8.1% vs 3.15%; p-value = 0.046], and [2.85% vs 11.29%; p-value < 0.001] respectively as shown (Table 1).

**Table 1:** Baseline characteristics of women with and without severe PPH after CD.

Variables	Severe PPH (n = 26)	No PPH (n = 702)	P value
Age category (n, %)			< 0.001*
< 20 years	2 (3.45)	56 (96.55)	
20–34 years	13 (2.26)	561 (97.74)	
≥ 35 years	11 (11.46)	85 (88.54)	
BMI (n, %)			0.42
< 24.9	3 (9.1)	30 (90.9)	
25–30	21 (3.1)	656 (96.9)	
> 30	2 (11.12)	16 (88.88)	
Parity (n, %)			0.74
1	19 (3.29)	557 (96.71)	
2–4	5 (4.54)	105 (95.46)	
≥ 5	2 (4.76)	40 (95.24)	
Place of residence (n, %)			0.89
Urban	9 (3.7)	234 (96.3)	
Rural	17 (3.5)	468 (96.5)	
Type of pregnancy (n, %)			0.046*
Singleton	21 (3.15)	645 (96.85)	
Twin	5 (8.1)	57 (91.9)	
Coexisting (n, %)			0.09
Yes	3 (8.82)	31 (91.18)	
No	23 (96.70	671 (3.3)	
ANC follow-up (n, %)			0.001*
Yes	19 (2.85)	647 (97.15)	
No	7 (11.29)	55 (88.71)	

*Statistical significant at p-value < 0.05, *BMI* body mass index.

### Obstetric characteristics of participants

In regards to obstetric characteristics, as shown in (Table 2), the severe PPH group was significantly associated with previous scar ≥ 2 (15.15% vs 4.12% vs 2.84%; p-value < 0.001), antepartum hemorrhage (17.24% vs 2.39%; p-value < 0.001), and severe preeclampsia (12.82% vs 3.05%; p-value = 0.001) compared those who did not develop SPPH.

**Table 2:** Obstetric characteristics with and without severe PPH after CD.

Variables	Severe PPH	No severe PPH	P value
Previous CS scar (n, %)			< 0.001*
0	16 (2.71)	574 (97.29)	
1	4 (4.12)	93 (95.88)	
≥ 2	6 (14.63)	35 (85.37)	
History of abortion (n, %)	4 (4.04)	95 (95.96)	0.39
History of AUB (n, %)	1 (8.34)	11 (91.66)	0.37
Prepartum anemia (Hgb < 11 g/dl) (n, %)			0.25
Yes	4 (6.06)	62 (93.94)	
No	22 (3.32)	640 (96.68)	
Failed induction/augmentation (n, %)	4 (3.28)	118 (96.72)	0.85
APH (n, %)			< 0.001*
Yes	10 (17.24)	48 (82.76)	
No	16 (2.39)	654 (97.61)	
Severe preeclampsia (n, %)			0.001*
Yes	5 (12.82)	34 (87.18)	
No	21 (3.05)	668 (96.95)	
Eclampsia (n, %)	1 (11.12)	8 (88.88)	0.22
HELLP syndrome	1 (16.67)	5 (83.33)	0.08
Prolonged labor (n, %)	4 (5.71)	66 (94.29)	0.31
Oligohydroaminous (n, %)	2 (6.9)	27 (93.1)	0.32
Polyhydroaminous (n, %)	1 (7.69)	12 (92.31)	0.42
PROM (n, %)	2 (2.56)	76 (97.44)	0.61
Preterm (n, %)	1 (2.78)	35 (97.22)	0.8
Failed instrumental vaginal birth (n, %)	1 (14.28)	6 (85.72)	0.63
Non-cephalic presentation. (n, %)	3 (3.37)	86 (96.63)	0.79
IUGR (n, %)	2 (5.26)	36 (94.74)	0.56

*Statistical significant at p-value < 0.05, *APH* antepartum hemorrhage, *PROM* premature rupture of membrane, *IUGR* intrauterine growth restriction.

### Perioperative characteristics of participants

Concerning perioperative characteristics, the severe PPH group was significantly associated with classic incision (42.86% vs 2.8%; p-value < 0.001), general anesthesia (19.6% vs 2.36%; p-value < 0.001), and stillbirth (14.1% vs 2.4%; p-value < 0.001) (Table 3).

**Table 3:** Perioperative characteristics with and without severe PPH after CD.

Variables	Severe PPH	No PPH	P value
Type of cesarean section (n, %)			0.12
Emergency	26 (3.88)	643 (96.12)	
Elective	0 (0)	59 (100)	
Incision type (n, %)			< 0.001*
Transverse	20 (2.82)	688 (97.18)	
Classic	6 (30)	14 (70)	
Type of anesthesia (n, %)			< 0.001*
General	12 (22.64)	41 (77.36)	
Spinal	14 (2.07)	661 (97.93)	
Sex of neonate (n, %)			0.94
Male	16 (3.62)	427 (96.38)	
Female	10 (3.51)	275 (96.49)	
Birth weight (n, %)			0.21
< 2500 g	6 (6.89)	81 (93.11)	
2500–4000 g	18 (3.18)	549 (96.82)	
> 4000 g	2 (2.7)	72 (97.3)	
Stillbirth (n, %)			< 0.001*
Yes	10 (14.1)	61 (85.9)	
No	16 (2.44)	641 (97.56)	

*Statistical significance at p-value < 0.05.

The bivariate analysis found that poor ANC follow-up, maternal age ≥ 35 years, previous scar ≥ 2, twin pregnancy, APH, severe preeclampsia, general anesthesia, classic incision, and stillbirth was significantly associated with severe PPH after CD.

### Factors associated with severe postpartum hemorrhage

The predictors found to be associated with severe PPH at bivariate analysis were further analyzed to control possible confounders. Multiple logistic regression analysis revealed that the likelihood of developing severe PPH after cesarean delivery among women diagnosed with severe preeclampsia was 4.52 times (AOR = 4.52, 95% CI 1.24‒16.46) higher compared to women without severe preeclampsia. Women presented with antepartum hemorrhage were 2.89 times (AOR 2.89, 95% CI 1.01‒8.16) as likely to have severe PPH compared to women without APH. Mothers ≥ 35 years of age were 2.77 times (AOR 2.77, 95% CI 1.02‒7.52) as likely to have severe PPH compared to their younger age groups. Women who had a history of CS scar ≥ 2 were 4.08 times (AOR 4.08, 95% CI 1.20‒13.86) more likely to develop severe PPH compared to their counterparts. The likelihood of developing severe PPH was 6.01 times (AOR 4.05; 95% CI 1.37‒11.95) higher among women who received general anesthesia than those who received spinal anesthesia. Moreover, classic incisions increase the risk of developing severe PPH 6-fold (AOR 6.01; 95% CI 1.51‒23.98) compared to transverse lower uterine segment resection (Table 4).

**Table 4:** Multivariable logistic regression analysis of independent predictors associated with severe PPH among women who underwent cesarean delivery.

Variables	Category	Severe PPH		COR (95% CI)	AOR (95% CI)	p-value
		Yes	No			
Severe preeclampsia	Yes	5	34	4.68 (1.66–13.17)	4.52 (1.24–16.46)	0.022*
	No	21	668	1	1	
Antepartum hemorrhage	Yes	10	48	6.23 (2.71–14.30)	2.89 (1.01–8.16)	0.042*
	No	16	654	1	1	
Age ≥ 35 years	Yes	11	85	5.32 (2.37–11.97)	2.77 (1.02–7.52)	0.045*
	No	15	617	1	1	
Twin pregnancy	Yes	5	57	2.69 (0.98–7.41)	3.19 (0.90–11.25)	0.071
	No	21	645	1	1	
CS scar ≥ 2	Yes	6	35	5.72 (2.16–15.13)	4.08 (1.20–13.86)	0.024*
	No	20	667	1	1	
Classic incision	Yes	6	14	14.74 (5.13–42.32)	6.01 (1.51–23.98)	0.011*
	No	20	688	1	1	
Poor ANC follow-up	Yes	19	647	4.34 (1.75–10.76)	2.99 (0.93–9.61)	0.065
	No	7	55	1	1	
General anesthesia	Yes	12	41	13.82 (6.1–31.8)	4.05 (1.37–11.95)	0.011*
	No	14	661	1	1	
Stillbirth	Yes	10	61	6.57 (2.86–15.10)	2.79 (0.92–8.49)	0.070
	No	16	641	1	1	

*Statistical significance at p-value < 0.05.

## Discussion

In this retrospective cohort study, we investigated the incidence and predictors of severe PPH among women who underwent cesarean delivery in the Southern Central Ethiopian population using detailed clinical data retrieved from 728 (97.58%) medical records of participants.

Our study demonstrated that the overall incidence of severe PPH after CS is 3.6%, modestly above the previously reported rate of 3 per 1000 mothers in the US^3^, 1.56% in China^5^, 1.2% in Uganda^6^, and 2.1% in Japan^8^. However, many studies^3–5,22–24^ depicted that cesarean birth poses an increased risk for severe PPH compared to vaginal delivery. In contrast, nationwide studies conducted in three different European countries found that the incidence of severe PPH (≥ 1000–2500 ml) ranged from 4.5 to 4.8%, which is slightly higher than our findings^11–13^. The wide variation in PPH definitions and management practice across the clinical setup and between countries could attribute to the observed discrepancies. Although comparing the complex health systems of high-income countries with resource-limited settings is odd; due to a lack of evidence on severe PPH after CS.

There are many possible reasons for the observed difference in the findings of the current and previous studies. First, unlike those studies, we only included women who underwent CD, a mode of delivery that increases the likelihood of severe PPH. Secondly; we utilized the definition of severe PPH using hemoglobin differences and units of blood transfused than the less accurate estimation of blood loss registered in medical records^29,30^. Variation between studies may also result from varied diagnosis approaches and medical interventions or healthcare systems. For example, the inaccessibility of tranexamic acid, fresh frozen plasma, and platelet concentrate^32^ may also contribute to the increased rate of severe PPH. Furthermore, the study setting was a referral hospital, where more complicated pregnancies were admitted from rural health centers of the catchment area. A recent nationwide retrospective study done in Japan also showed that cases of PPH managed in large referral hospitals increased over time^34^.

The current study demonstrated that severe preeclampsia, CS scar ≥ 2, antepartum hemorrhage, maternal age ≥ 35 years, vertical incision, and general anesthesia were risk factors associated with severe PPH among women who underwent CD.

The study observed that mothers presented with severe preeclampsia were 4.14 times more likely to develop severe PPH. The finding is in line with many studies^3,8^. This could be due to the multifactorial pathogenesis of preeclampsia, resulting in hypertension and coagulation abnormalities to cause bleeding that evolves into severe PPH.

Mothers presented with previous CS scar ≥ 2 were 4.08 times more likely to have severe PPH when compared to mothers with one and no cesarean scar. Findings from other studies^7,26,27^ also revealed that the likelihood of developing severe PPH is significantly higher in mothers with the previous scar than in their counterparts. This indicates that the presence of CS scar might predispose them to significant bleeding and uterine atony due to the formed adhesion and increased risk of abnormal placentation.

Mothers with advanced age ≥ 35 years were 2.77 times more likely to develop severe PPH among mothers who underwent cesarean delivery (AOR 2.77; 95% CI (1.02–7.52). This finding agrees with many studies conducted in different clinical setups^3,4,21^. This could be because obstetric complications and interventions increase as maternal age increases to^35^.

Antepartum hemorrhage (APH) mothers were 4.8 times more at risk of developing severe PPH. This finding is consistent with other studies^5,24,27,28^ that depicted the risk of developing severe PPH as higher in mothers with APH. In the risk prediction model of women undergoing CD, women with APH (abruption on presentation) had a threefold risk for requiring peripartum blood transfusion due to severe PPH^25^.

We found a strong association between severe PPH and types of anesthesia (AOR 4.05, 95% CI (1.37–11.95), the odds of mothers who received general anesthesia were 4.05 times more likely to have severe PPH as compared to the odds of mothers who received spinal anesthesia, in agreement with other studies^23,24,28^. This might be due to more complicated pregnancies like severe preeclampsia, APH with active bleeding, and hemodynamically unstable mothers needing critical care tend to receive general anesthesia than spinal anesthesia. Moreover, inhalational anesthetic agents have a concentration-dependent inhibitory effect on uterine contraction, thereby increasing the chance of uterine atony^36^. In a population-based study of 67,328 women who had live singleton births by CS, the odds of experiencing cesarean PPH with general anesthesia are approximately 8.15 times higher than with epidural anesthesia^37^. Thus, the impact of anesthesia types on the risk of developing PPH needs further investigation.

When the lower uterine segment incision is not enough to provide adequate access for delivery, midline vertical incisions (classical CS), are alternatively considered. Compared to lower-segment incision, the classical incision is associated with much more blood loss and a significant reduction of maternal hemoglobin^38^. Our study also found that mothers who underwent classical CD were 3.6 times more at risk for severe PPH compared to low incisions. This indicates that in cases of vertical incision, more vascular and thicker myometrial tissue may be surgically incised, thereby increasing the likelihood of severe bleeding. Given these facts, the guideline minimizes the possibility of using vertical high-incision, proposing this procedure only in selected cases when there is anatomical difficulty in accessing the lower uterine segment.

Generally, high-risk women anticipated to develop severe PPH should be managed at an advanced center with well-organized obstetric and anesthesiology care and a consistent blood supply for transfusion^39^. Therefore, multidisciplinary skilled team participation is crucial in the development of management pathways that are customized institutionally and should comprise the creation of guidelines that are anesthesia specific, including protocols for obstetrics and operating units, at large^40^.

## Strengths and limitations

Unsurprisingly, estimating blood loss during CS is more accurate than other modes of delivery. However, numerous studies have found that clinicians underestimate the volume lost, making accurate assessment more challenging. The main strength of this study is the diagnosis and definitions of severe PPH were determined using hemoglobin change and the need for blood transfusion, which is more reliable and accurate compared to estimated blood loss. Despite its precision as retrospectively assessed, using the value of hemoglobin difference before and after CS and the requirement for blood transfusion might delay the diagnosis of severe PPH as the clinicians need to wait for the blood test results. The study might not only be limited to detecting an ongoing clinical deterioration attributed to underestimating the extent of bleeding but also limited to rapidly responding to deterioration. In another way, the retrospective nature of the data and a focus exclusively concerns severe postpartum hemorrhage in the setting of in-labor CS, resulting in perhaps failure to predict similar complications that occur amongst vaginal and elective cesarean deliveries. Furthermore, known risk factors, including birth interval and mothers with a previous history of PPH, were not included in the analysis since > 60% of study participants were referred from rural health institutions where their previous medical records are unknown. This may explain the need for future prospective observational studies that included the missed predictors to extrapolate the findings in daily clinical practice.

## Conclusion

The incidence of severe PPH after cesarean delivery was relatively similar to other studies. Severe preeclampsia, antepartum hemorrhage, advanced age ≥ 35 years, previous CS scar ≥ 2, classic incision, and general anesthesia were significantly associated factors with severe PPH after CS. Based on our findings, we recommend considering appropriate uterotonic agents and less invasive hemostatic interventions, which could help reduce the rate of severe PPH and its related morbidity.

## Data Availability

The datasets used and/or analyzed during the current study are available from the corresponding author upon reasonable request.
